# Blocking phosphatidylglycerol degradation in yeast defective in cardiolipin remodeling results in a new model of the Barth syndrome cellular phenotype

**DOI:** 10.1016/j.jbc.2021.101462

**Published:** 2021-12-02

**Authors:** Paulína Káňovičová, Petra Čermáková, Dominika Kubalová, Lenka Bábelová, Petra Veselá, Martin Valachovič, Jakub Zahumenský, Anton Horváth, Jan Malínský, Mária Balážová

**Affiliations:** 1 Department of Membrane Biochemistry, Institute of Animal Biochemistry and Genetics, Centre of Biosciences, Slovak Academy of Sciences, Bratislava, Slovakia; 2 Department of Biochemistry, Faculty of Natural Sciences, Comenius University, Bratislava, Slovakia; 3 Department of Functional Organization of Biomembranes, Institute of Experimental Medicine, Academy of Sciences of the Czech Republic, Prague, Czech Republic

**Keywords:** phosphatidylglycerol, tafazzin, mitochondria, Barth syndrome, valproic acid, BTHS, Barth syndrome, Erg1, squalene epoxidase, Erg11, lanosterol 14-alpha-demethylase, ETS capacity, maximum electron transfer system capacity, CL, cardiolipin, Crd1, cardiolipin synthase, DAG, diacylglycerol, MLCL, monolysocardiolipin, OXPHOS capacity, oxidative phosphorylation capacity, PA, phosphatidic acid, PC, phosphatidylcholine, PE, phosphatidylethanolamine, PG, phosphatidylglycerol, Pgc1, phosphatidylglycerol phospholipase C, PGP, phosphatidylglycerol phosphate, Pgs1, phosphatidylglycerolphosphate synthase, PI, phosphatidylinositol, PS, phosphatidylserine, RCI, respiratory control index, SE, sterol esters, TAG, triacylglycerols, Taz1, lyso-phosphatidylcholine acyltransferase, VPA, valproic acid, WT, wild type

## Abstract

Barth syndrome (BTHS) is an inherited mitochondrial disorder characterized by a decrease in total cardiolipin and the accumulation of its precursor monolysocardiolipin due to the loss of the transacylase enzyme tafazzin. However, the molecular basis of BTHS pathology is still not well understood. Here we characterize the double mutant *pgc1*Δ*taz1*Δ of *Saccharomyces cerevisiae* deficient in phosphatidylglycerol-specific phospholipase C and tafazzin as a new yeast model of BTHS. Unlike the *taz1*Δ mutant used to date, this model accumulates phosphatidylglycerol, thus better approximating the human BTHS cells. We demonstrate that increased phosphatidylglycerol in this strain leads to more pronounced mitochondrial respiratory defects and an increased incidence of aberrant mitochondria compared to the single *taz1*Δ mutant. We also show that the mitochondria of the *pgc1*Δ*taz1*Δ mutant exhibit a reduced rate of respiration due to decreased cytochrome *c* oxidase and ATP synthase activities. Finally, we determined that the mood-stabilizing anticonvulsant valproic acid has a positive effect on both lipid composition and mitochondrial function in these yeast BTHS models. Overall, our results show that the *pgc1*Δ*taz1*Δ mutant better mimics the cellular phenotype of BTHS patients than *taz1*Δ cells, both in terms of lipid composition and the degree of disruption of mitochondrial structure and function. This favors the new model for use in future studies.

Mitochondria represent a highly specialized and dynamic functional organelle comprised of two structurally distinct lipid bilayers, the inner and outer mitochondrial membranes. Two negatively charged phospholipids, phosphatidylglycerol (PG) and cardiolipin (CL) are highly specific for mitochondria. CL is an atypical phospholipid with four acyl chains, which exhibits numerous functions in mitochondrial bioenergetics and biogenesis (1). PG is an obligatory CL precursor and usually a low-abundance phospholipid. Outside mitochondria, PG fulfills essential roles in specific membranes of some eukaryotic organisms (2, 3). For example, it is the preferred phospholipid in thylakoid membranes of photosynthetic organisms (4) or an important component of the pulmonary surfactant in mammals (5, 6). In addition, there is a growing body of evidence that PG can, at least partially, substitute some functions of CL when it is missing (7), or that it may in fact be PG itself that performs some of the functions originally attributed to CL (8, 9). In general, a decrease in mitochondrial CL content at the expense of PG or other anionic lipids is the most frequently described pathological change in CL profile associated with a large number of diseases (10).

Final steps of CL biosynthesis include remodeling of the immature CL molecule. The newly synthesized CL is first converted to monolyso-CL (MLCL) and subsequently reacylated to mature CL by a transacylase or acyltransferase (11). This way, an optimal, tissue-specific fatty acid composition of CL is achieved. Mutations in the MLCL transacylase tafazzin, encoded by the *TAZ* gene, were identified as the primary cause of the X chromosome-linked recessive disease called Barth syndrome (BTHS). Clinically, BTHS is characterized by abnormal mitochondria, dilated cardiomyopathy, neutropenia, skeletal myopathy, growth delay, exercise intolerance, and increased level of organic acid in the urine (7, 8). As a result of *TAZ* mutations, BTHS patients exhibit decreased levels of CL and accumulate MLCL in mitochondria. Besides that, both PG and CL of these patients contain reduced amounts of linoleic acid (C18:2), a characteristic acyl chain found in mature mammalian CL (12).

Several relevant studies have demonstrated the importance of the yeast *Saccharomyces cerevisiae* as a model for studying lipid biosynthesis disorders, including BTHS. Steps of CL synthesis are highly evolutionarily conserved; therefore, the yeast *taz1*Δ mutant has been used as a simple model to study defects resulting from altered remodeling and deficiency of CL (13). Indeed, heterologous expression of the human *TAZ* gene was sufficient to rescue the phenotype of *taz1*Δ yeast cells (14), which made the yeast BTHS model attractive for studies focused on molecular mechanisms underlying the BTHS pathology. There is however a remarkable difference between human BTHS and yeast *taz1*Δ cells, a significantly lower PG content in the latter (12). The amount of PG in wild-type yeast is at the limit of detection, even under conditions of increased CL biosynthesis during active respiration (15, 16), and it is not much increased in the absence of Taz1 (9, 13). This aspect could be of crucial importance, as recent data indicated that PG levels *per se* affect mitochondrial morphology and function (16, 17). Low levels of PG thus reduce the reliability of the yeast *taz1*Δ mutant as a plausible BTHS model, as it cannot be ruled out that the relatively higher PG participates in generating a complex BTHS phenotype in mammals.

PG level in yeast is controlled by PG-phosphate (PGP) synthase Pgs1 and PG-specific phospholipase Pgc1 through an effective mechanism capable of fast, wide-range, and bidirectional PG regulation (18). Deletion of *PGC1* leads, under conditions of sustained Pgs1 activity, to nonspecific accumulation of PG without distinct side effects on the amounts of other phospholipids, including CL, but with apparent adverse effects on mitochondrial fusion and respiration (16). Therefore, in this study, we tested whether the deletion of *PGC1* in *taz1*Δ cells could generate a yeast BTHS model that would better simulate the PG/CL ratio detected in mammalian cells.

As an alternative approach to regulate PG levels in the yeast BTHS model, we tested the effect of valproic acid (VPA) treatment on the analyzed yeast strains. VPA is a broad-spectrum antiepileptic drug that has been widely used for more than 60 years and is approved by the Food and Drug Administration (FDA) for the treatment of bipolar disorders and neuralgia. Although the mechanism of its therapeutic effect is not yet clear, it is known that among other effects, VPA inhibits *de novo* synthesis of inositol from glucose-6-phosphate by indirectly blocking *myo*-inositol phosphate synthase (19). In yeast, inositol inhibits PGP synthase, Pgs1, catalyzing the rate-limiting step of PG *de novo* synthesis. Accordingly, increased biosynthesis of PG and CL in response to VPA treatment has been reported in yeast during fermentation (20). Here we report that VPA changes the content of anionic phospholipids in yeast grown on nonfermentable carbon source only moderately, but is capable of restoring the coupling between the electron transport and ATP synthesis, affected in *taz1*Δ and *pgc1*Δ*taz1*Δ mutants.

## Results

### Phospholipid characterization of pgc1Δtaz1Δ double mutant

In humans, the cellular PG content is in general higher compared with yeast (12, 21). Following the *TAZ* gene disruption, PG levels in human cells further increase (21). Under conditions of sustained production, in principle there are two ways how to increase the generally low PG content in yeast—either its utilization as a CL precursor or its direct degradation can be compromised. Accumulation of PG in a yeast strain lacking the CL synthase Crd1 rescued some defects caused by the loss of CL (7). Deletion of *PGC1* gene coding for PG-specific phospholipase C (15) resulted also in PG accumulation, but at normal CL levels in mitochondria (16). Therefore, to prepare a yeast BTHS model with an elevated PG content, we deleted *PGC1* gene in *taz1Δ* strain. All the experiments were performed in media without inositol to stimulate PGP synthase, Pgs1 (22, 23), and with nonfermentable carbon sources, to stimulate mitochondrial activity.

**Figure 1:**
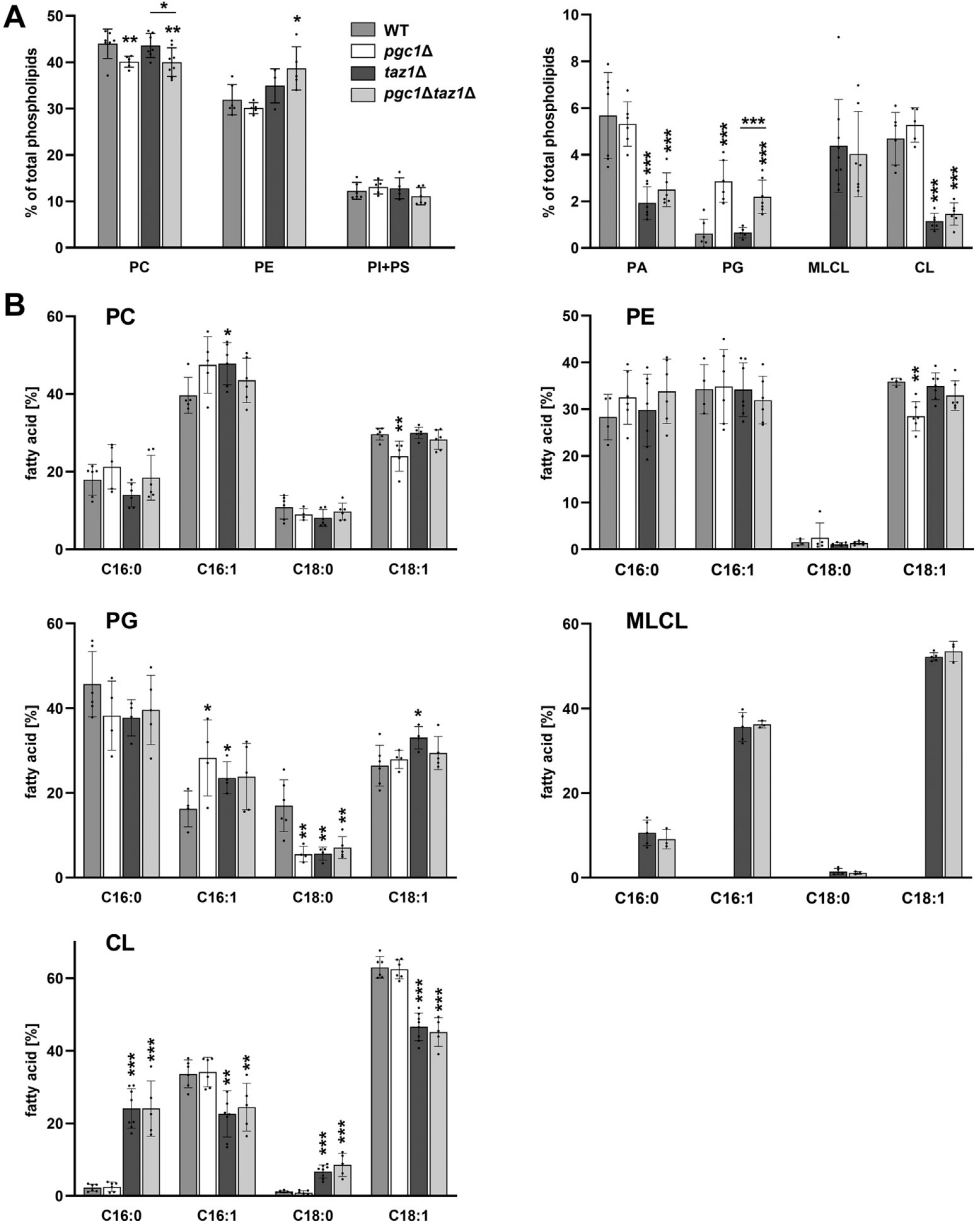
**Compared with *taz1*Δ, *pgc1*Δ*taz1*Δ strain contains an increased level of PG.** Wild type, *pgc1*Δ, *taz1*Δ, and *pgc1*Δ*taz1*Δ strains of *S. cerevisiae* (see Table 1 for details) were cultivated in SMDGE I- medium for 24 h. Lipids from mitochondrial fractions were extracted, separated, and relative amounts of phospholipids were calculated based on the contents of inorganic phosphate (*A*). In mitochondrial fractions of analyzed strains, PC, PG, CL, and MLCL fatty acids were identified by the gas chromatography (*B*). MLCL content was determined in *taz1*Δ and *pgc1*Δ*taz1*Δ strains only. In the other two strains, negligible MLCL amounts were detected. Data represent mean values from a minimum of five independent experiments ±SEM. Statistically significant differences between mutant strains and the wild type, or between *pgc1*Δ*taz1*Δ and *taz1*Δ strain are marked. ∗*p* < 0.05; ∗∗*p* < 0.01; ∗∗∗*p* < 0.001. CL, cardiolipin; MLCL, monolysocardiolipin; PA, phosphatidic acid; PC, phosphatidylcholine; PE, phosphatidylethanolamine; PG, phosphatidylglycerol; PI, phosphatidylinositol; PS, phosphatidylserine; WT, wild type.

As expected, mitochondria of the *pgc1Δtaz1Δ* mutant exhibited a combined lipid composition phenotype: (i) similar to the *taz1Δ* strain, the defect in CL remodeling led to the MLCL accumulation in the double deletion strain and (ii) the absence of Pgc1 resulted in increased levels of PG in this strain, probably mainly at the expense of PC fraction (Fig. 1*A*). Besides the PG accumulation and PC depletion, no statistically significant difference between the *taz1Δ* and *pgc1Δtaz1Δ* strains was detected.

Previously, we reported differences between the fatty acid composition of PG accumulated in *pgc1*Δ and *crd1*Δ strains (16). Similarly to these strains, the acyl chain composition of mitochondrial PG in *taz1Δ* significantly differed from the wild type (Fig. 1*B*). Specifically, in PG isolated from the mitochondria of *taz1*Δ cells, we found increased palmitoleic acid (C16:1; 145 ± 30% of the wild type value) and oleic acid (C18:1; 125 ± 14% of the wild-type value). This increase was fully at the expense of stearic acid (C18:0; 33 ± 10% of the wild-type value), which was similar to the changes detected in *pgc1*Δ and *pgc1Δtaz1Δ* strains. Consistent with previously published data (9, 13), the CL-bound fatty acids content in the analyzed strains depended solely on Taz1 activity, as *pgc1*Δ profile was indistinguishable from wild type, and similarly, *pgc1Δtaz1Δ* profile was comparable with *taz1Δ* strain. In the latter two strains, we observed a pronounced decrease in unsaturated palmitoleic (C16:1) and oleic acid (C18:1) and an increase in saturated palmitic (C16:0) and stearic acid (C18:0) bound to CL molecules. Similarly, the fatty acid composition of MLCL was not affected by the further deletion of *PGC1* gene (Fig. 1*B*).

### Sterols and sterol esters in pgc1Δtaz1Δ double mutant

Besides defects in CL remodeling, decreased cholesterol synthesis has been observed in lymphoblasts of BTHS patients after serum starvation (24, 25). Therefore, we tested levels of ergosterol and its derivatives in the yeast mutants mimicking BTHS.

**Figure 2:**
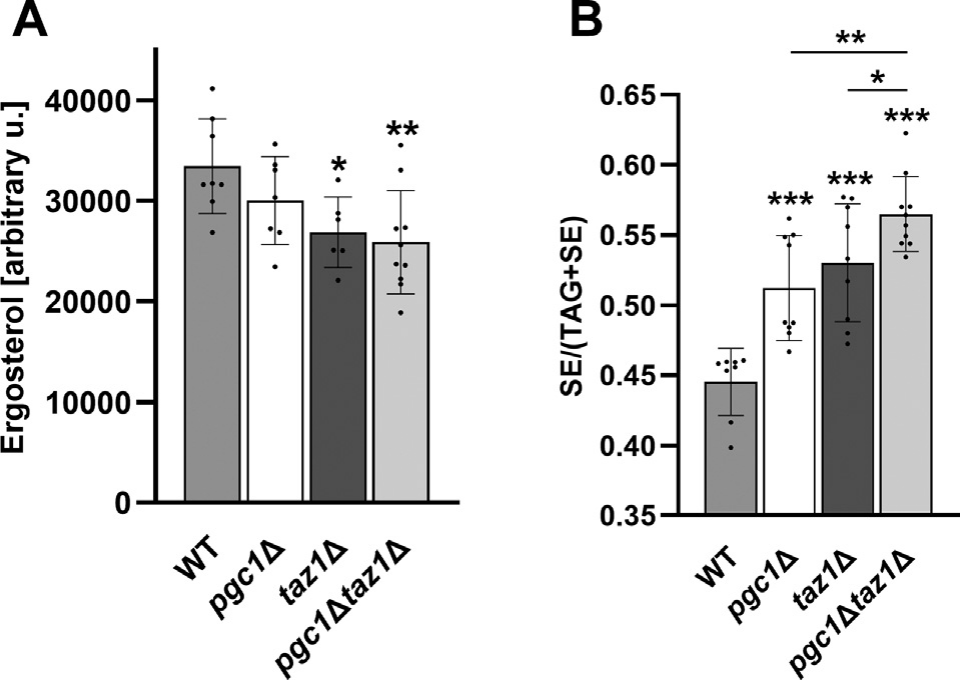
***TAZ1* and/or *PGC1* deletions affect ergosterol metabolism.** Wild type, *pgc1*Δ, *taz1*Δ, and *pgc1*Δ*taz1*Δ strains (see Table 1 for details) were cultivated in SMDGE I- medium for 24 h. Yeast homogenates prepared by zymolyase treatment were used for neutral lipid extraction. Extracted lipids were separated by TLC and scanned at 193 nm. Ergosterol content (*A*) and a relative fraction of SE in total neutral lipids (TAG+SE; *B*) were determined. Data represent mean values from eight independent experiments ±SEM. Statistically significant differences between mutant strains and wild type, or *pgc1*Δ*taz1*Δ and *taz1*Δ strain, or *pgc1*Δ*taz1*Δ and *pgc1*Δ strain are marked. ∗*p* < 0.05; ∗∗*p* < 0.01; ∗∗∗*p* < 0.001. SE, sterol esters; TAG, triacylglycerol; WT, wild type.

We compared the neutral lipid content in wild type, *pgc1*Δ, *taz1*Δ, and *pgc1*Δ*taz1*Δ strains. Significantly decreased ergosterol levels in both strains lacking *TAZ1* gene were detected. Approx. 20% drop of ergosterol content was detected in these strains independent of the presence of Pgc1 (Fig. 2*A*). The analysis also revealed an increased fraction of sterol esters (SE) in the neutral lipids of all deletion mutants analyzed. In both single deletion mutants, SE fraction was significantly overrepresented if compared with the wild type. Importantly, this fraction further increased in *pgc1*Δ*taz1*Δ cells (Fig. 2*B*), suggesting different mechanisms of SE elevation in each of the single mutants.

### Effect of elevated PG on mitochondrial morphology and function in pgc1Δtaz1Δ cells

Phospholipid composition of mitochondrial membranes affects the organelle morphology and function. In cells and tissues of BTHS patients, abnormal mitochondrial ultrastructure and increased mass of mitochondria were observed (26, 27, 28, 29). Similarly in yeast *taz1*Δ mutant, aberrant cristae morphology and swollen mitochondria have been observed (9, 30). To further characterize the *pgc1*Δ*taz1*Δ strain, we compared mitochondria of this strain with those of the wild type, *pgc1*Δ, and *taz1*Δ strains.

**Figure 3:**
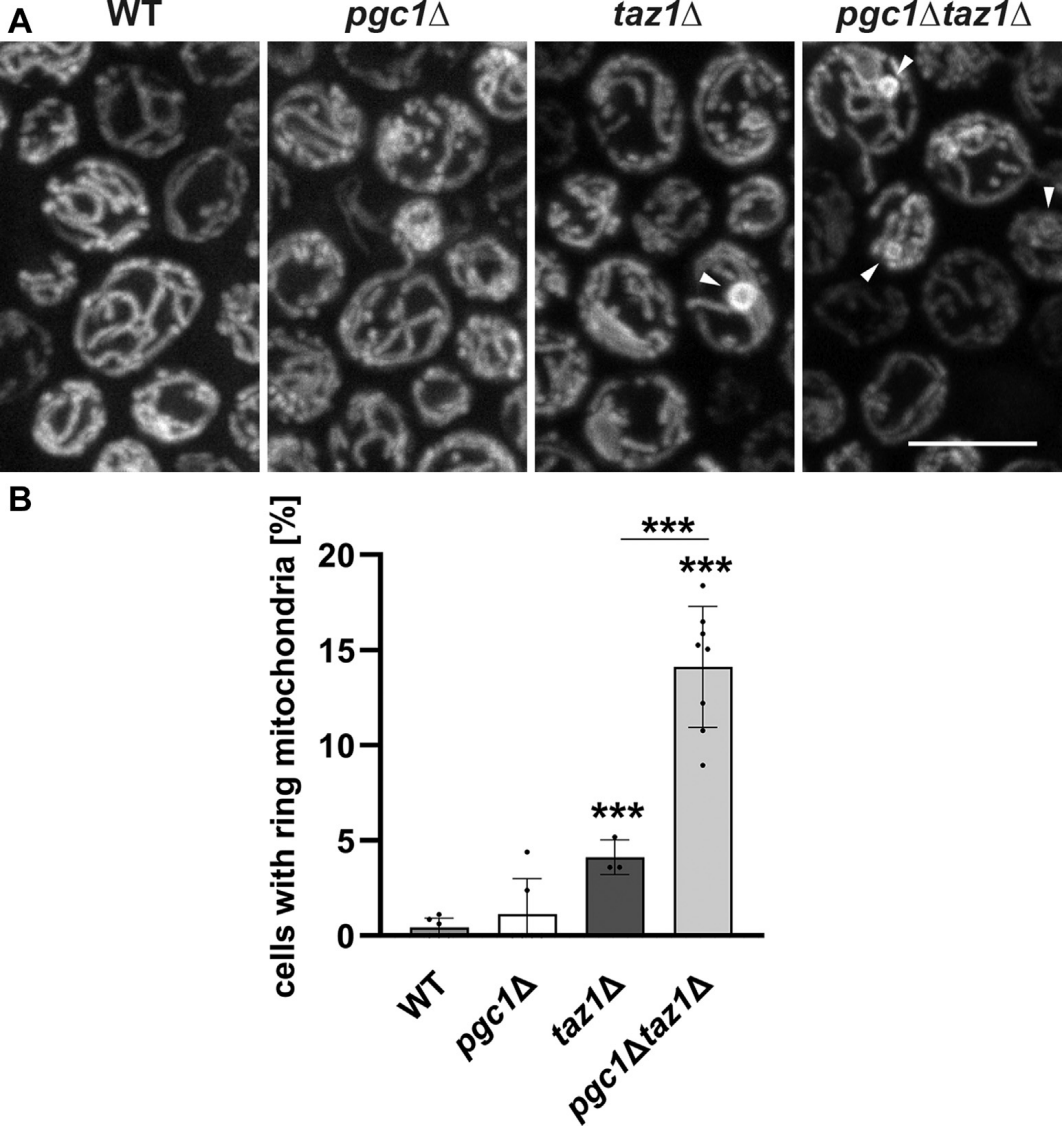
**Deletion of *PGC1* increases occurrence of morphologically aberrant mitochondria in *taz1*Δ cells.** Mitochondrial morphology was visualized by Mitotracker Red staining in wild type, *pgc1*Δ, *taz1*Δ, and *pgc1*Δ*taz1*Δ cells grown in SMDGE I- medium for 24 h. Maximum intensity projections of six consecutive confocal sections are presented (*A*). Abnormal, ring-shaped mitochondria were detected in mutants lacking *TAZ1* gene (*arrowheads*). The occurrence of ring mitochondria was quantified in each strain (*B*). Sets of 200 cells were analyzed in three biological replicates of the experiment. Statistically significant differences between mutant strains and the wild type or *pgc1*Δ*taz1*Δ and *taz1*Δ strain are marked. Bar: 5 μm. ∗∗∗*p* < 0.001. WT, wild type.

**Figure 4:**
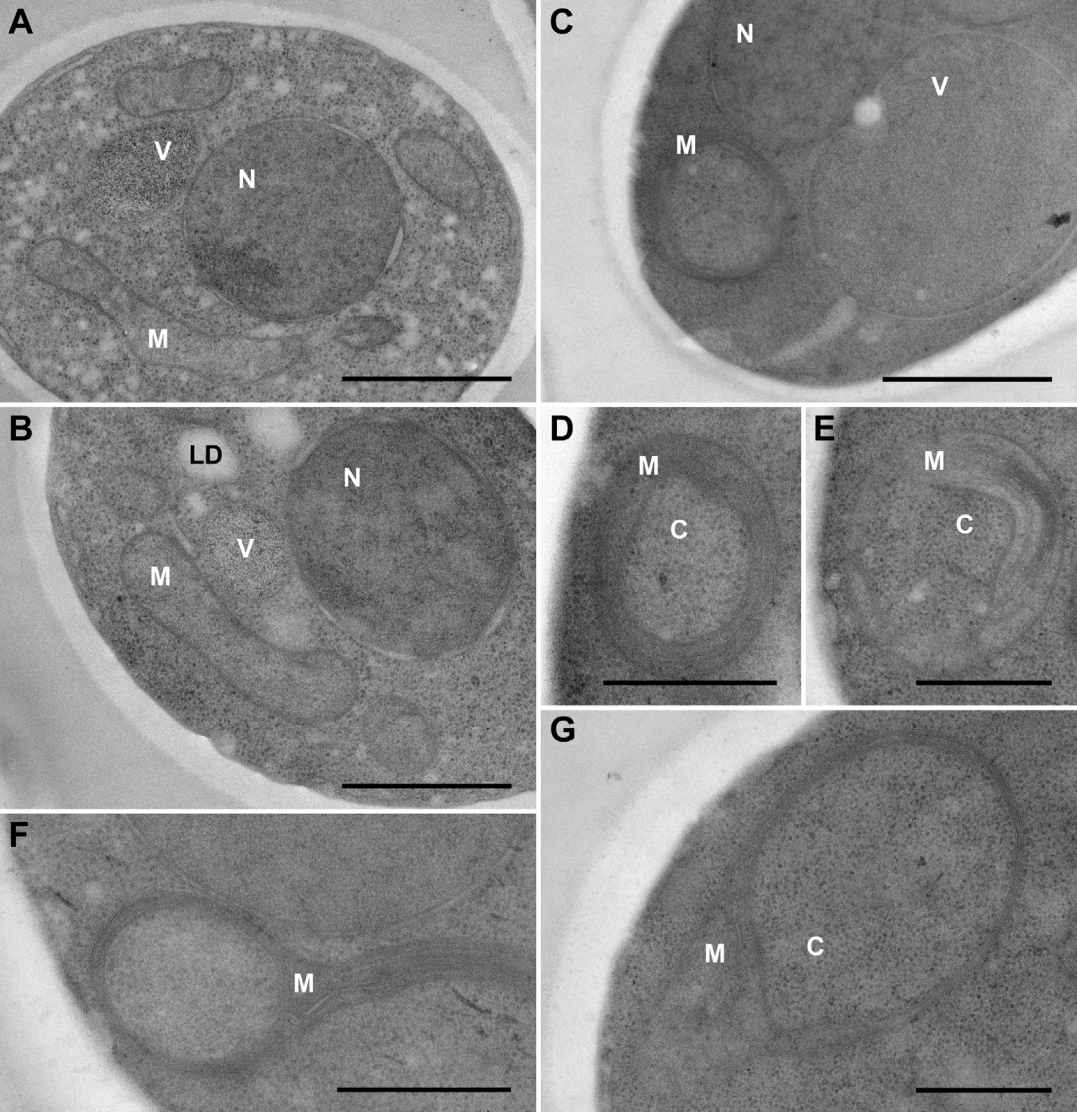
**Mitochondria in *pgc1*Δ*taz1*Δ mutant represent two populations of differential fine structure.** Ultrathin sections of *pgc1*Δ*taz1*Δ cells imaged by transmission electron microscopy are presented. Normal, rod-like mitochondria (*A* and *B*) and aberrant, ring-shaped mitochondria (*C*–*G*) are shown in cellular context (*A*–*C*) and in detail (*D*–*G*). Relevant cellular compartments are marked in all figures. Bars: 1 μm (*A*–*C*), 500 nm (*D*–*G*). C, cytoplasm; LD, lipid droplet; M, mitochondrion; N, cell nucleus; V, vacuole.

To do this, we cultivated wild type, *pgc1*Δ, *taz1*Δ, and *pgc1*Δ*taz1*Δ yeast in SMDGE medium without inositol for 24 h and visualized mitochondria *in situ* using Mitotracker-Red fluorescent dye (Fig. 3*A*). As expected, aberrant mitochondria were occasionally observed in *taz1*Δ cells. The aberrant mitochondria were of a characteristic ring shape. The frequency of this aberration was almost tripled in the double deletion strain (Fig. 3, *A* and *B*). As apparent at the ultrastructural level, the aberrant mitochondria contained excessive amounts of juxtaposed extended membranes/cristae. At the same time, the morphology of “normal,” rod-like mitochondria of the same strain did not significantly differ from the wild type (Fig. 4), with the following exception: deletion of *PGC1* gene in wild-type strain increased mitochondrial fragmentation as we also reported previously (16). In accordance with this observation, a similar tendency could be recognized when mitochondrial morphology of *taz1*Δ and *pgc1*Δ*taz1*Δ strains were compared.

The morphology of mitochondria is tightly connected with mitochondrial functionality. For example, the aforementioned mitochondrial fragmentation in *pgc1*Δ cells was accompanied by decreased coupling between electron transport and ATP synthesis (16). Similarly, the formation of aberrant, flat mitochondrial sheets correlated with extensive respiratory defects of the *crd1*Δ strain lacking cardiolipin synthase. These included suboptimal respiratory chain function due to destabilization of respiratory chain supercomplexes (7, 9, 16, 31). Finally, MLCL accumulation and CL reduction in *taz1*Δ mutant resulted not only in emergence of aberrantly shaped mitochondria but also in increased respiratory rates and decreased respiratory control index (RCI), a measure of the coupling between the electron transport and ATP synthesis in the analyzed mitochondria (9, 14). Therefore, we tested the functional relevance of the increased frequency of ring-shaped mitochondria in *pgc1*Δ*taz1*Δ strain.

**Figure 5:**
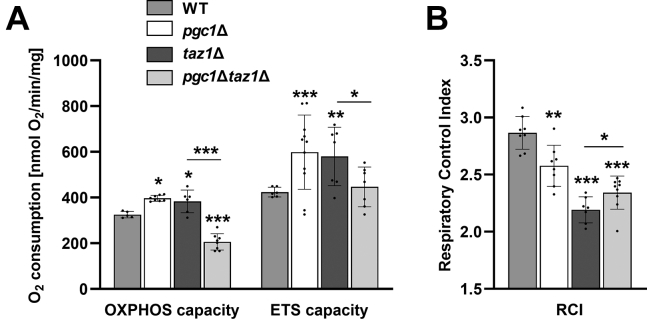
**Mitochondrial respiration is slowed down by the combined *PGC1* and *TAZ1* deletion.** Yeast cells were cultivated in SMDGE I- media for 24 h. Isolated mitochondria were used to measure oxygen consumption. NADH was used as a respiratory substrate. Mitochondrial respiration was measured in the presence of ADP (OXPHOS capacity) and protonophore CCCP (ETS capacity) (*A*). Respiratory control index was measured (*B*). Data represent mean values from six independent experiments in two technical replicates ±SEM. Statistically significant differences between mutant strains and wild type or *pgc1*Δ*taz1*Δ and *taz1*Δ strain are marked. ∗*p* < 0.05; ∗∗*p* < 0.01; ∗∗∗*p* < 0.001. WT, wild type.

Respiratory capacity was measured in isolated mitochondria of the wild type, *pgc1*Δ, *taz1*Δ, and *pgc1*Δ*taz1*Δ strains in the ADP-activated state, in the presence of NADH (OXPHOS capacity), and after uncoupling of mitochondria with CCCP (maximum electron transfer system capacity, ETS capacity; Fig. 5*A*). In single deletion mutants, both OXPHOS and ETS capacity were increased compared with wild type. In the double deletion strain, the OXPHOS capacity was reduced to almost half of the wild-type value. The ETS capacity did not significantly differ from the wild type. This result indicated severe impairment of respiratory activity in the double mutant mitochondria compared with single mutants *taz1*Δ and *pgc1*Δ. Consistent with previously published data (9, 16), we detected significantly decreased RCI in both *pgc1*Δ and *taz1*Δ strains, indicating weaker coupling between the electron transport and ATP synthesis. Double mutant *pgc1*Δ*taz1*Δ exhibited slightly higher RCI than *taz1*Δ (Fig. 5*B*).

**Figure 6:**
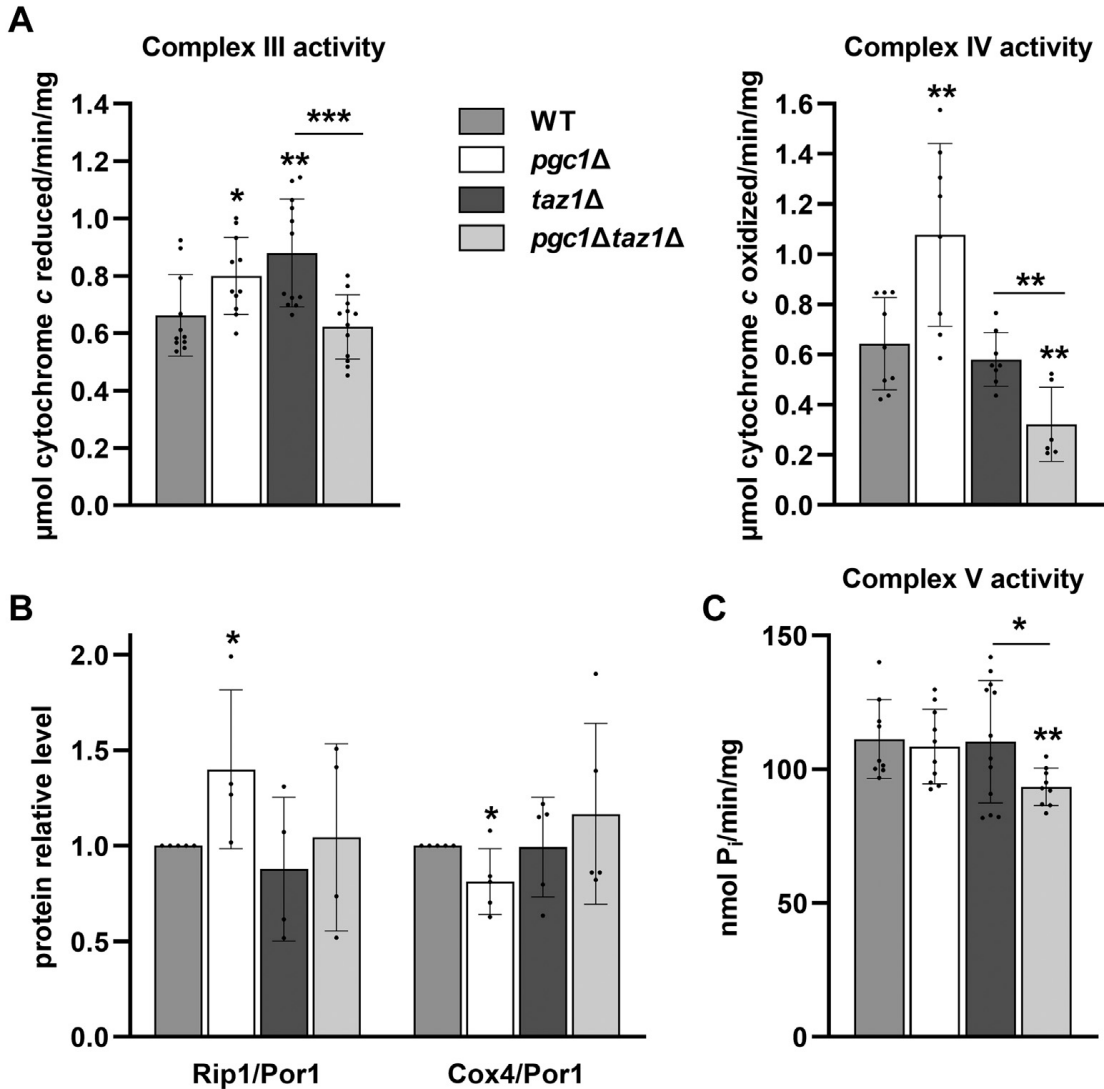
**Deletion of *PGC1* decreases the activity of respiratory complexes III, IV, and V in *taz1*Δ strain.** Lysates prepared from isolated mitochondria of the wild type, *pgc1*Δ, *taz1*Δ, and *pgc1*Δ*taz1*Δ yeast (see Experimental procedures for details) were used to measure the activities of respiratory complexes. *In vitro* activity of Complex III and Complex IV (*A*). Relative levels of Rip1 (subunit of Complex III) and Cox4 (subunit of Complex IV) were normalized to Por1 level (*B*). *In vitro* activity of Complex V (*C*). Data represent mean values from 4 to 5 independent experiments ±SEM. Statistically significant differences between mutant strains and wild type or *pgc1*Δ*taz1*Δ and *taz1*Δ strain are marked. ∗*p* < 0.05; ∗∗*p* < 0.01; ∗∗∗*p* < 0.001. WT, wild type.

Next, we measured individual *in vitro* activities of cytochrome *c* reductase, cytochrome *c* oxidase, and ATP synthase (Complexes III, IV, and V, respectively) in isolated mitochondria. Measurements of Complex III activity revealed increased cytochrome *c* reduction in mitochondria of *pgc1*Δ and *taz1*Δ strains compared with the wild type. Complex III activity in double mutant *pgc1*Δ*taz1*Δ was comparable with the wild type (Fig. 6*A*). The increased activity in single deletion strains corresponds with the observed increased OXPHOS capacity and ETS capacity (Fig. 5*A*). Interestingly, we detected different amounts of Complex III subunit Rip1 in *pgc1*Δ and *taz1*Δ strains. While increased Rip1 expression could have stood behind the increased Complex III activity in *pgc1*Δ strain, *taz1*Δ mitochondria achieved an even higher activity with wild-type Rip1 levels (Fig. 6*B*). The activity of Complex IV was reduced to half of the wild-type level in mitochondria of *pgc1*Δ*taz1*Δ (Fig. 6*A*). This was probably the reason for the decrease in OXPHOS capacity in this strain (Fig. 5*A*). It is noteworthy that we found the normal level of Complex IV subunit Cox4 in mitochondria of the double mutant (Fig. 6*B*). Finally, a small, but significant decrease in Complex V activity compared with the wild type was detected also in the double deletion mutant (Fig. 6*C*).

Our results identified significant changes in respiration efficiency resulting from *PGC1* deletion in *taz1*Δ cells. On the one hand, it was capable to compensate for an increased Complex III activity in *taz1*Δ cells. On the other hand, it reduced the activity of Complex IV and Complex V.

### Regulation of PG level in Taz1-deficient cells

As we documented above, the absence of Taz1 leads, among other effects, to a substantial reduction in the level of mitochondrial CL (Fig. 1). It has been published that the role of CL in mitochondrial functions can be partially substituted by PG (7, 32). Therefore, any imbalance of PG level in Taz1-deficient cells should directly affect respiratory chain performance. To check how the PG level is controlled in these cells, we compared the activities of PGP synthase Pgs1 and PG-specific phospholipase C Pgc1 in the respective strains.

**Figure 7:**
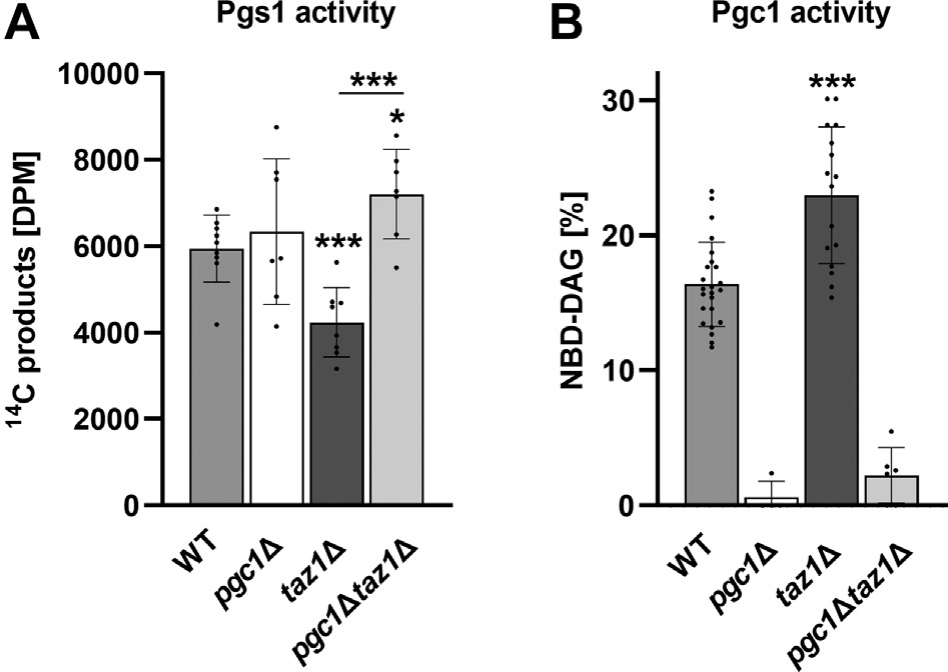
**Low level of PG is tightly controlled in *taz1*Δ strain.** Wild type, *pgc1*Δ, *taz1*Δ, and *pgc1*Δ*taz1*Δ cells were cultivated in SMDGE I- medium for 24 h. *In vitro* PGP synthase (Pgs1) activity was analyzed in isolated mitochondrial fraction after the addition of the radioactive substrate (see Experimental procedures for details; *A*). *In vitro* phospholipase activity of Pgc1 in cell homogenate prepared by zymolyase treatment was measured in terms of relative amounts of NBD-DAG, a fluorescent product of Pgc1-mediated hydrolysis of NBD-PG (*B*). Data represent mean values from five independent experiments in two technical replicates ±SEM. Statistically significant differences between mutant strains and wild type or *pgc1*Δ*taz1*Δ and *taz1*Δ strain are marked. ∗*p* < 0.05; ∗∗*p* < 0.01; ∗∗∗*p* < 0.001. WT, wild type.

*In vitro* measurements on mitochondria isolated from the wild type, *pgc1*Δ, *taz1*Δ, and *pgc1*Δ*taz1*Δ cells revealed a reduced activity of Pgs1 in *taz1*Δ strain. In contrast, Pgc1 activity in this strain was higher compared with the wild type. In other words, despite the comparable PG levels in the wild type and *taz1*Δ cells (Fig. 1), mitochondria of the mutant strain exhibited a decreased capacity for PG production, but an increased capacity for PG degradation (Fig. 7, *A* and *B*). In accordance with our expectations, only residual PG degradation was detected in *pgc1*Δ*taz1*Δ cells, similar to the single deletion mutant *pgc1*Δ (Fig. 7*B*). Surprisingly, the Pgs1 activity was fully restored in *pgc1*Δ*taz1*Δ cells – in fact, we detected even slightly increased activity compared with the wild type (Fig. 7*A*).

### Effect of valproic acid on taz1Δ and pgc1Δtaz1Δ phenotypes

The activity of PGP synthase Pgs1 is inhibited by inositol. Consequently, drop in inositol supply leads, among other effects, to increased PG production (20). Mood-stabilizing anticonvulsant VPA is known to deplete intracellular inositol levels (33, 34). Accordingly, in yeast logarithmically growing on glucose, radioactive labeling of phospholipids revealed increased steady-state production of CL in cells treated with 0.6 mM VPA (20). Therefore, we tested whether VPA treatment can influence CL and/or PG levels also under conditions of intensive respiration during the growth on nonfermentable carbon source and specifically, whether these changes could be enough to affect the *taz1*Δ phenotype.

The analyzed strains, wild type, *pgc1*Δ, *taz1*Δ, and *pgc1*Δ*taz1*Δ, were grown in SMDGE medium in the absence of inositol and presence of 0.006, 0.06, and 0.6 mM VPA. In accordance with a previous study (20), significant retardation of cell growth was observed in all strains treated with 0.6 mM concentration of VPA (Fig. S1), suggesting that such a dose of VPA induced severe changes in the cellular metabolism. Some of the VPA effects include the impact on ergosterol biosynthesis or antifungal sensitivity. More specifically, we observed increased tolerance to fluconazole and accumulation of ergosterol precursor lanosterol in cells treated with 0.6 mM VPA (Fig. S2).

**Figure 8:**
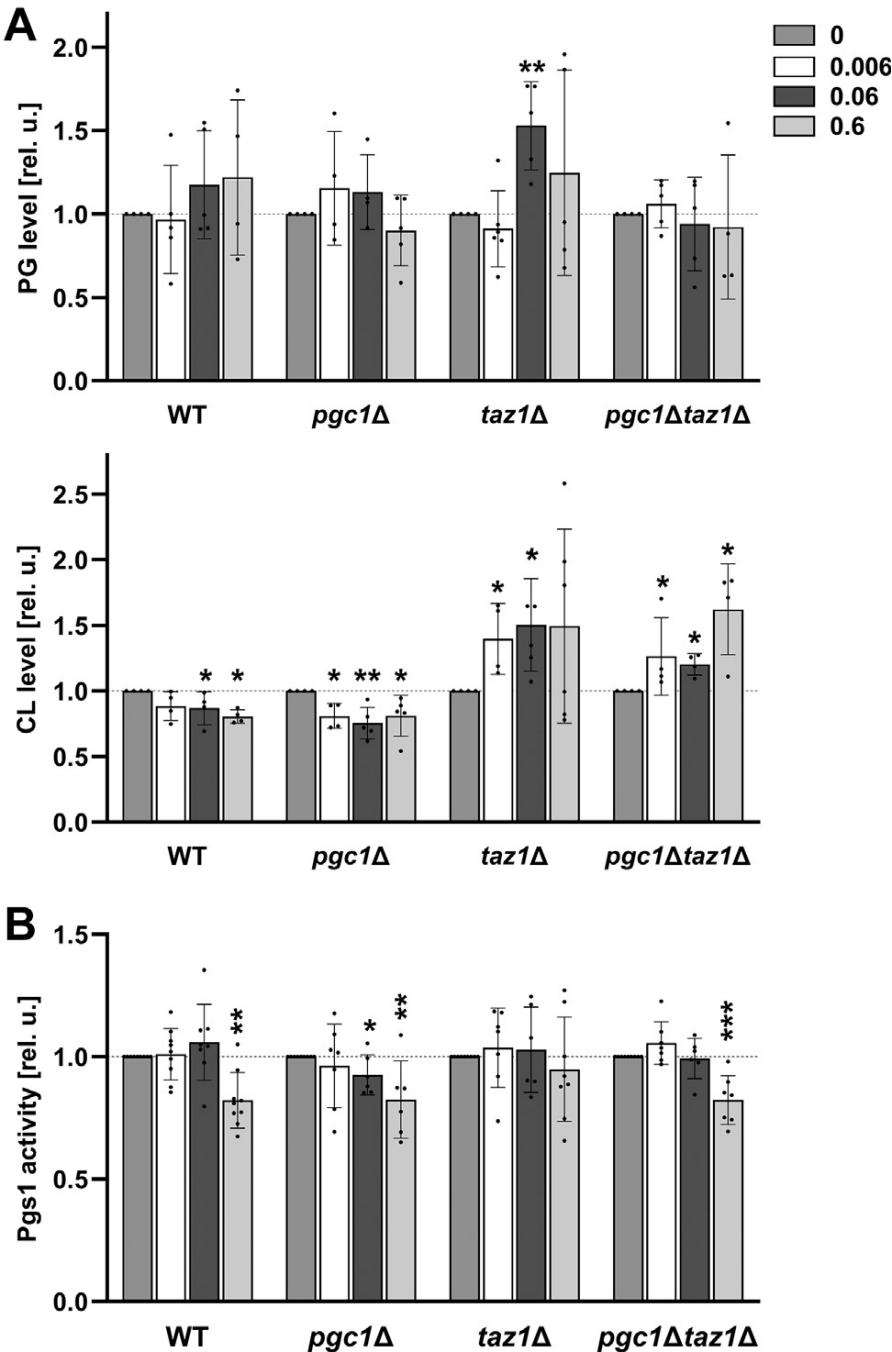
**VPA has no substantial effect on CL biosynthesis in conditions of intense respiratory growth.** Wild type, *pgc1*Δ, *taz1*Δ, and *pgc1*Δ*taz1*Δ cells (see Table 1 for details) were cultivated in SMDGE I- medium for 24 h following the addition of 0, 0.006, 0.06, or 0.6 mM VPA. Lipids from mitochondrial fractions were extracted, separated, and analyzed. Relative amounts of PG and CL were calculated (*A*). Relative levels of *in vitro* Pgs1 activity measured in isolated mitochondria were calculated, too (*B*). All data were normalized to the levels detected in untreated samples (samples with 0 mM VPA). Data represent mean values from a minimum of five independent experiments ±SEM. Statistically significant differences between 0.006, 0.06, 0.6 and 0 mM VPA concentration are marked. ∗*p* < 0.05; ∗∗*p* < 0.01; ∗∗∗*p* < 0.001. CL, cardiolipin; PG, phosphatidylglycerol; WT, wild type.

However, even the application of lower VPA concentrations led to statistically significant alterations of the CL levels in mitochondria of all the analyzed strains. Specifically, in cells containing *TAZ1* gene (*i.e.*, cells of the wild type and *pgc1*Δ strains) we observed decreased and in those lacking *TAZ1* allele increased levels of CL following the VPA treatment. Additionally in *taz1*Δ strain, PG level was increased (Fig. 8*A*). High VPA concentrations also attenuated Pgs1 activity (Fig. 8*B*). Below the limit of statistical significance were VPA-induced changes in *in vitro* degradation of PG by the phospholipase Pgc1 (not shown). We conclude that under selected conditions, VPA slightly increases CL production in strains with impaired CL remodeling.

**Figure 9:**
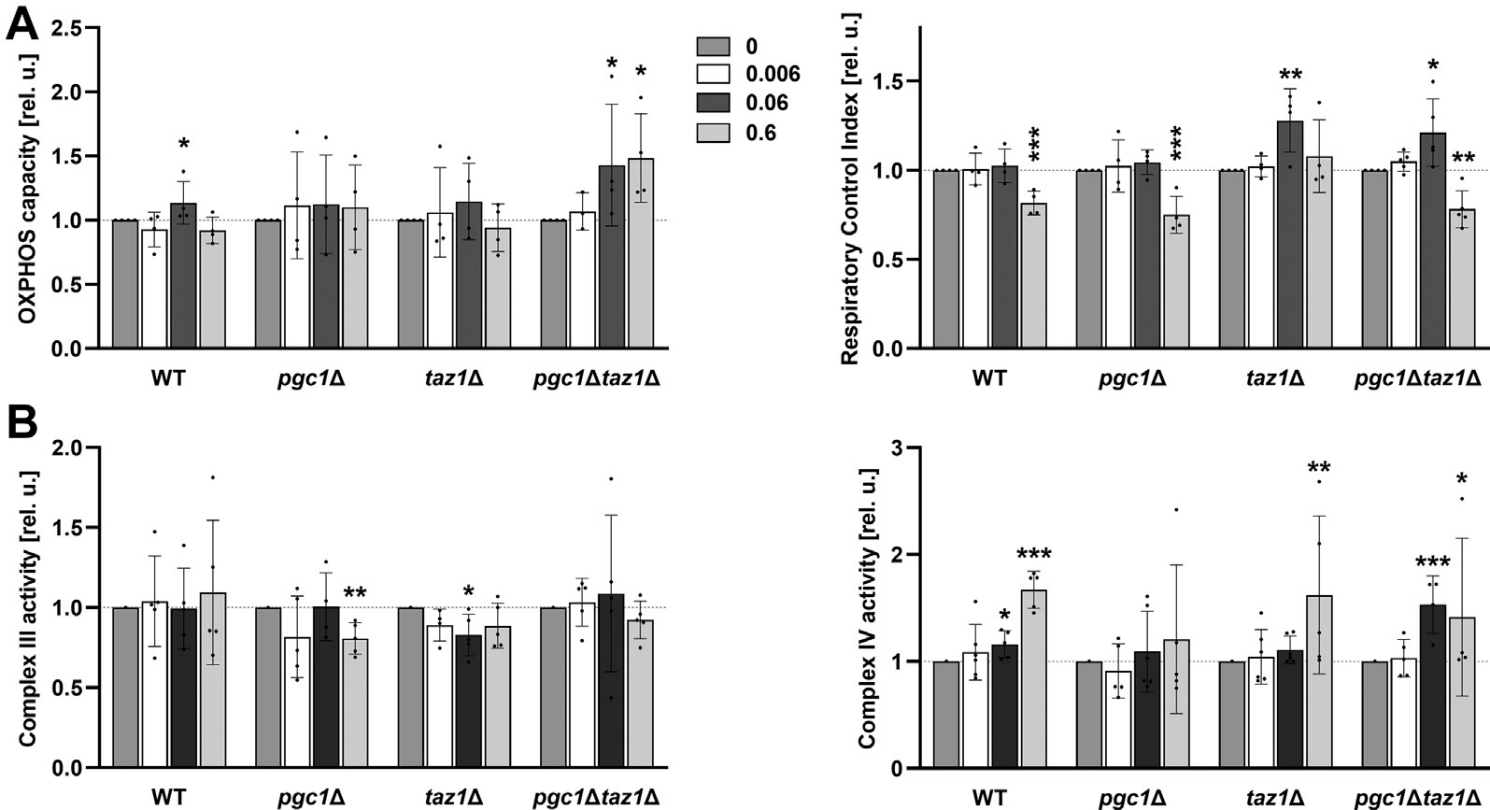
**VPA treatment affects mitochondrial respiration.** Yeast cells were cultivated in SMDGE I- media for 24 h with the addition of 0, 0.006, 0.06, or 0.6 mM VPA. Isolated mitochondria were used to measure oxygen consumption. NADH was used as a respiratory substrate. Relative levels of mitochondrial respiration in the presence of NADH and ADP (OXPHOS capacity; *A*, *left panel*), respiratory control index as a measure of OXPHOS coupling (*A*, *right panel*), and Complex IV and III activities (*B*, *left* and *right panel*, respectively) are presented. All data were normalized to the levels detected in untreated samples (samples with 0 mM VPA). Data represent mean values from minimal four independent experiments in two replicates ±SEM. Statistically significant differences between 0.006, 0.06, 0.6 and 0 mM VPA concentration are marked. ∗*p* < 0.05; ∗∗*p* < 0.01; ∗∗∗*p* < 0.001. WT, wild type.

As shown in Figure 5*A*, the rate of O_2_ consumption in mitochondria isolated from *pgc1*Δ*taz1*Δ cells was slowed down to about one-half of the wild-type value. Interestingly, the cultivation of *pgc1*Δ*taz1*Δ cells with 0.06 mM VPA increased the OXPHOS capacity in these mitochondria almost by 50% (Fig. 9*A*). Similarly, a significant increase of RCI could be observed in both strains lacking *TAZ1* after treatment with 0.06 mM VPA. In contrast, the application of higher VPA concentration decreased RCI in three of four analyzed strains including the wild type, supportive to the generally observed growth defect in 0.6 mM VPA-treated cells (Fig. S1).

The beneficial effect of 0.06 mM VPA on mitochondrial respiration of cells defective in CL remodeling was further analyzed by comparing the changes in the activity of respiration complexes III and IV following the VPA treatment in all studied strains. A pronounced increase of Complex IV activity has been detected in *pgc1*Δ*taz1*Δ mitochondria following the treatment with 0.06 mM VPA. This was in clear contrast to the isolates from the other analyzed strains (wild type and the two single deletion mutants), in which the increase was at or even below the verge of statistical significance. In all strains treated with 0.6 mM VPA, we observed large increases in the activity of complex IV, the statistical significance of which was greatly exacerbated by the large variance between the individual samples analyzed (Fig. 9*B*, right panel).

In contrast to Complex IV, the activity of Complex III was not strongly affected by treatment with 0.06 mM VPA. The only significant change was the slight decrease of Complex III activity detected in *taz1*Δ strain (Fig. 9*B*, left panel). This imbalance of Complex III and IV activities indicated a reduction in respiratory coupling between electron transfer and ATP synthesis, induced by VPA treatment. It is noteworthy here that the direct addition of VPA to isolated mitochondria of untreated *pgc1*Δ*taz1*Δ cells, even at a concentration of 0.6 mM, did not affect their respiratory functions (not shown). Apparently, the effect observed on isolated mitochondria of VPA-treated cells reflected VPA-induced changes in cellular metabolism rather than the direct effect of VPA binding to any of the mitochondrial components.

## Discussion

This study has been motivated by the effort to prepare a realistic yeast model of BTHS. Being aware of the fact that compared with yeast, human cells contain higher levels of PG, we addressed the role of PG elevation in the manifestation of the BTHS phenotype in yeast. As expected, mitochondria of the newly constructed double deletion mutant, *pgc1Δtaz1Δ*, exhibited a phospholipid profile, which combined the effects of both single mutations—decreased CL content with MLCL accumulation caused by the *TAZ1* deletion and elevated PG due to *PGC1* deletion. The increased amount of PE detected in the *pgc1*Δ*taz1*Δ strain (Fig. 1*A*) can be interpreted as a positive compensatory effect of the missing CL, as both PE and CL tend to incorporate into negatively curved membranes. This way PE in concert with PG could structurally substitute CL in respiration complexes and compensate for some CL functions, as described elsewhere (35, 36). Supportive of this interpretation, we observed a decreased abundance of unsaturated acyl chains in (unremodeled) CL fractions of both *taz1Δ* and *pgc1Δtaz1Δ* strains (Fig. 1*B*) as was previously described in BTHS patients (37). Apparently, not only the decreased CL amount but also its improper fatty acid composition contributed to the phenotypes observed in these mutants.

Besides changes in acyl chain composition of CL, we also detected a pronounced decrease in stearate (C18:0) and an increase in palmitoleate (C16:1) fractions of mitochondrial PG in all analyzed mutant strains (Fig. 1*B*). For *pgc1Δ* strain, we reported this observation earlier (16). Finding that deletion of *TAZ1* gene leads to a similar change in PG acyl chain profile is not easy to interpret. No additive effect of simultaneous deletion of *PGC1* and *TAZ1* was detected, however. It suggested that changes observed in both single mutants either reached saturation or they resulted from the same origin. Shift to shorter and unsaturated acyl chains increases membrane fluidity in the mutant mitochondria. Among other possible causes for this change in phospholipid composition, it could reflect the overall decrease of ergosterol content in the membranes of the mutant cells (Fig. 2*A*). In mixed lipid membranes, sterols function as a solvent of highly ordered lipids (38). Ergosterol depletion could thus be functionally corrected by such a phospholipid adaptation.

Our observation, that in *taz1*Δ and *pgc1*Δ*taz1*Δ strains cultivated in medium containing nonfermentable carbon source ergosterol are depleted, is in an agreement with earlier studies that reported decreased levels of cholesterol in cells of BTHS patients under conditions of serum starvation (24, 25). Although the decrease observed in *pgc1*Δ strain was not statistically significant (Fig. 2*A*), the *pgc1*Δ cells exhibited, similar to *taz1*Δ and *pgc1*Δ*taz1*Δ mutants, a significant increase in sterol ester fraction (Fig. 2*B*). The pronounced additive effect of the double *PGC1* and *TAZ1* genes deletion, observed in this case, suggests that if the defect in ergosterol biosynthesis is somehow related to the (saturated) effect on PG acyl chain composition, then it lies upstream of it. SE, together with triacylglycerols, is stored in lipid droplets. Therefore, increased SE fraction in all the analyzed mutant strains, indicating a connection between PG and CL biosynthesis and lipid droplets, is not surprising. We showed before that Pgc1 localizes predominantly onto lipid droplets although the protein is active in membranes of endoplasmic reticulum and mitochondria (18). Increased lipid storage has been described as a frequent complication in *TAZ1*-deficient BTHS patients (39, 40, 41).

Accumulation of PG affects mitochondrial morphology and function. It has been reported that mitochondria of *pgc1*Δ cells, which accumulate PG at normal CL levels, are more fragmented compared with the wild type, and those of *crd1*Δ cells, which accumulate PG in the absence of CL, form large sheets. Moreover, the frequency of mitochondrial sheets increased in *pgc1*Δ*crd1*Δ cells, which accumulated even more PG compared with the single mutants (16). In accordance with this, we detected an increased frequency of aberrant, ring-shaped mitochondria in *pgc1*Δ*taz1*Δ cells, if compared with *taz1*Δ strain (Figs. 3 and 4). The occurrence of ring-shaped mitochondria in yeast cells lacking *TAZ1* gene corresponds well with earlier detection of “onion-shaped” mitochondria with collapsed cristae arranged in concentric layers that have been described in lymphoblasts of BTHS patients (27, 42, 43).

Mitochondrial defects as increased rate of oxygen consumption, decreased RCI, and destabilization of respiratory protein supercomplexes have been previously described in yeast *taz1*Δ mutant (9, 14, 44). Our data showed that the deletion of *PGC1* in these cells resulted in normalization of ETS capacity and partial increase of RCI, but at the cost of a massive decrease of OXPHOS capacity (Fig. 5). The latter effect was probably caused by a significant reduction in Complex IV activity in this strain. This loss of cytochrome *c* oxidase activity was detected together with a normal amount of Cox4 in *pgc1*Δ*taz1*Δ mitochondria (Fig. 6, *A* and *B*), which was consistent with their normal ETS capacity. It seems that the primary reason for the decreased OXPHOS capacity in *pgc1*Δ*taz1*Δ mutant was a decreased activity of Complex V (Fig. 6*C*). This interpretation is supported by the observation of more frequent aberrations of cristae morphology in these cells, indicating affected ATP synthase dimers-containing inner mitochondrial membrane (Fig. 4) (45).

We found that PG content is tightly controlled in *taz1*Δ cells. The potentially negative effect of PG accumulation is prevented through both the downregulation of PG synthesis and increased degradation in this mutant. Interestingly enough, this self-protection failed in *pgc1*Δ*taz1*Δ cells. Under conditions of absent PG degradation, the double mutant could not suppress the activity of PGP synthase (Fig. 7).

It has been published before that during fermentation, the activity of PGP synthase, an enzyme catalyzing the critical step of CL biosynthesis, can be increased by VPA (20). In this study, we show that also under conditions of respiratory growth (nonfermentable carbon source, absence of extracellular inositol, diauxic growth phase), VPA treatment led to an increase of CL content in cells defective in CL remodeling (Fig. 8*A*). This observation positively correlates with the increase of RCI in *taz1*Δ and *pgc1*Δ*taz1*Δ mitochondria following the treatment with 0.06 mM VPA (Fig. 9, *A* and *B*, respectively). Together these findings indicate that VPA partially recovered respiration in these strains. However, these changes are not accompanied by a significant modulation of Pgs1 activity (Fig. 8*B*), suggesting that another mechanism of CL elevation took place here. We can only speculate that, for example, VPA facilitated the use of unremodeled CL in the respiratory chain of these cells.

The growth defect recognized in all yeast strains treated with 0.6 mM VPA (Fig. S1) indicated that the observed alterations of mitochondrial function induced by VPA could not be specifically attributed to the regulation of PG/CL biosynthetic pathway performance. All cells treated with high concentrations of VPA also exhibited changes in ergosterol biosynthesis. Although VPA treatment did not affect the ergosterol levels in membranes of the treated cells, it significantly inhibited ergosterol synthesis at the lanosterol demethylation step. In addition, we observed the suppression of the inhibitory effect of antifungal fluconazole (Fig. S2). This indicates that VPA could act on the level of Erg11, the enzyme responsible for the initial step in the conversion of lanosterol to zymosterol (46, 47, 48). This is not surprising, since VPA is a known inhibitor of human cytochrome P450 isoforms (49, 50), and Erg11 is a yeast member of the cytochrome P450 family (51). An earlier finding that VPA synergistically interacted with another antifungal agent, terbinafine, in *Candida albicans* may provide further support for this interpretation (52). Terbinafine inhibits squalene epoxidase, Erg1, an enzyme upstream of Erg11 (53).

Altogether the presented data show that cells with impaired CL remodeling are highly sensitive to PG content. In the newly designed yeast strain *pgc1*Δ*taz1*Δ, elevated PG exacerbated the characteristic phenotype of *taz1*Δ cells: it increased the frequency of aberrant mitochondria and the respiration deficiency. This finding is consistent with the earlier reported detrimental effect of PG accumulation on mitochondrial structure and function (16). Most importantly, elevated PG makes the *pgc1*Δ*taz1*Δ more suitable model for mimicking the situation in tafazzin-deficient mammalian cells. We also documented that, to some extent, defects of *taz1*Δ and *pgc1*Δ*taz1*Δ cells could be suppressed by the VPA treatment. Application of this finding to mammalian BTHS models will be a subject of further studies.

## Experimental procedures

### Yeast strains and growth conditions

All *S. cerevisiae* strains used in this study are listed in Table 1. Cell cultures were grown in complex media YPD (2% peptone, 1% yeast extract, 2% glucose). For experiments, yeasts were grown aerobically at 30 °C for 24 h to diauxic shift in a defined synthetic SMDGE medium prepared as previously described (54), with 0.2% glucose, 3% glycerol, and 1% ethanol as a carbon source. SMDGE medium lacked inositol (I-). During VPA treatment, cells were cultivated for 24 h without or with the addition of 0.006, 0.06, or 0.6 mM sodium valproate (Sigma-Aldrich).

**Table 1:** Yeast strains

Strain	Genotype	Source
BY4741, wild type (WT)	*MAT*a *his3Δ1 leu2Δ0 met15Δ0 ura3Δ0*	Euroscarf
*pgc1*Δ	BY4741; *pgc1::KanMX4*	Euroscarf
*taz1*Δ	BY4741; *taz1::KanMX4*	Euroscarf
*pgc1*Δ*::NatMX4*	BY4741; *pgc1::NatMX4*	This study
*pgc1*Δ*taz1*Δ	BY4741; *taz1::KanMX4, pgc1::NatMX4*	This study

All *S. cerevisiae* strains were constructed in BY4741 background (Euroscarf).

### Strains construction

Mutant strain *pgc1*Δ*::NatMX4* was prepared by replacement of *pgc1::KanMX4* disruption cassette with *pgc1::NatMX4* as described in (55). Double mutant strain *pgc1*Δ*taz1*Δ was prepared as follows: a disruption cassette *taz1*Δ*::KanMX4* was prepared using chromosomal DNA isolated from *taz1*Δ strain as a template and using forward primer 5′-GGT ACA GCA TAA TCA ATG GTA GC-3′ and reverse primer 5′-GCC TTG ACC TCA TTT TCT ACT AAC-3′. The obtained PCR product was transformed into *pgc1*Δ*::NatMX4* strain. Yeast transformation was performed by the lithium acetate method (56).

### Fluorescence microscopy

Yeast cell culture grown in SMDGE I- medium for 24 h at 30 °C and stained with Mitotracker Red CMX-Ros (Thermo Fisher Scientific) as described (16). Stained cells were concentrated by brief centrifugation, immobilized on a 0.17 mm cover glass by a thin film of 1% agarose prepared in 50 mM potassium phosphate buffer (pH 6.3) and observed using LSM 880 (Zeiss) laser scanning confocal microscope with 100× PlanApochromat oil-immersion objective (NA = 1.4). Fluorescence signal of Mitotracker Red (excited by 561 nm line of solid state laser) was detected using bandpass 578 to 696 nm emission filter. Maximum intensity projections were calculated with ImageJ software (ImageJ, U. S. National Institutes of Health).

### Transmission electron microscopy

Yeast cell culture grown in SMDGE I- medium for 24 h at 30 °C was processed as described previously (57). Briefly: cells were concentrated by suction filtration, loaded in a flat specimen carrier, and quickly frozen in Leica EM PACT high-pressure freezer. Frozen samples were freeze substituted in acetone supplemented with 3% glutaraldehyde (EMS; 10% stock in acetone), 0.1% uranyl acetate (Polysciences; 20% methanolic stock), 1% OsO_4_ (EMS; 10% stock in acetone), and 1% water in Leica AFS machine and then embedded in Lowicryl HM20 resin (EMS). Ultrathin sections (70 nm) were cut with Ultracut S ultramicrotome equipped with a diamond knife (45◦; Diatome) and placed on copper formvar-coated grids. Sections were examined in an FEI Morgagni 268(D) transmission electron microscope at 80 kV. Images were captured with Mega View G2 CCD camera (Olympus).

### Mitochondrial enzymatic assays

Yeast cells were grown in SMDGE I- medium for 24 h to diauxic shift. Intact mitochondria were isolated as described previously (16). The final mitochondrial pellet was suspended in the respiration buffer (0.6 M mannitol, 20 mM HEPES/KOH pH 7.1, 2 mM MgCl_2_, 1 mM EGTA, 0.1% fatty-acid-free bovine serum albumin, 10 mM KH_2_PO_4_) and used for measurement of O_2_ consumption, the activity of cytochrome *c* reductase, the activity of cytochrome *c* oxidase, and ATP-hydrolase activity as described in (16) and measurement of Pgc1 activity as described in (18).

PGP synthase activity was determined by quantification of the incorporation of radiolabeled substrate [^14^C]glycerol-3-phosphate into chloroform-soluble products as described in (16, 58) with some modifications. The PGP synthase assay was performed in the presence of 50 mM MES-HCl pH 7.0, 0.1 mM MnCl_2_, 0.083 mM CDP-DAG, 1 mM Triton X-100, 0.02 mM [^14^C]glycerol-3-phosphate (40,000 CPM/nmol), and mitochondrial fraction corresponding to 25 μg of mitochondrial protein in a total volume of 120 μl for 20 min at 30 °C. The reaction was stopped by the addition of 1.5 ml chloroform:methanol:HCl mixture (100:100:0.6), followed by the addition of 1 ml of water to produce two phases. Aliquots of 0.45 ml of the organic layer were transferred into scintillation vials and evaporated under the stream of nitrogen. A scintillation mixture (2 ml) was added, and the radioactivity of each sample was determined with a scintillation counter.

### Neutral lipid analysis

Lipids from homogenate fraction prepared by Zymolyase treatment (corresponding to 3 mg of proteins) were extracted, dried under nitrogen stream, resuspended in chloroform:methanol mixture (2:1), and separated by thin-layer chromatography (TLC) as described (59). Relative lipid content was determined using CAMAG WinCATS software after scanning TLC plates on CAMAG TLC scanner 3 at 193 nm.

### Miscellaneous

Phospholipid analysis was performed as described previously (18), fatty acids analysis, and Western blot analysis as described in (16). Statistical comparisons were carried out by one-way analysis of variance using SigmaPlot 12 software (Systat Software). All graphs (GraphPad Prism 9 software, GraphPad Software) show the mean ± SD.

## Data availability

All data are contained within the manuscript and supporting information.

## Supporting information

This article contains supporting information (60, 61).

## Conflict of interest

The authors declare that they have no conflicts of interest with the contents of this article.
