# Supplementary material for: Blocking phosphatidylglycerol degradation in yeast defective in cardiolipin remodeling results in a new model of the Barth syndrome cellular phenotype
Source: J Biol Chem. 2021 Dec 2;298(1):101462. doi: 10.1016/j.jbc.2021.101462 (PMC8728584; doi:10.1016/j.jbc.2021.101462)
Supplement: Supplemental Figures S1 and S2 [file mmc1.pptx]

## Slide 1
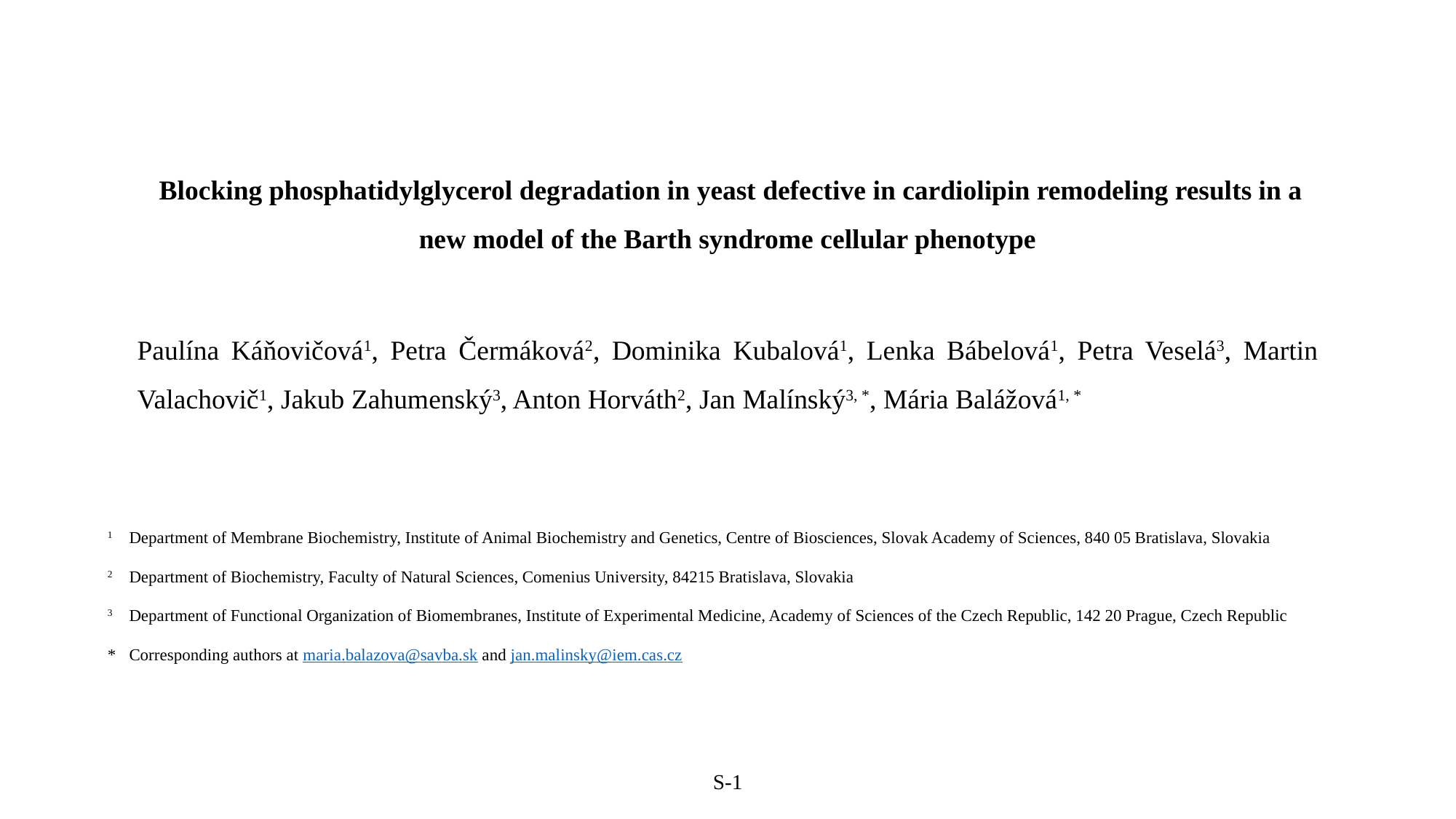

Blocking phosphatidylglycerol degradation in yeast defective in cardiolipin remodeling results in a new model of the Barth syndrome cellular phenotype
Paulína Káňovičová1, Petra Čermáková2, Dominika Kubalová1, Lenka Bábelová1, Petra Veselá3, Martin Valachovič1, Jakub Zahumenský3, Anton Horváth2, Jan Malínský3, *, Mária Balážová1, *
1	Department of Membrane Biochemistry, Institute of Animal Biochemistry and Genetics, Centre of Biosciences, Slovak Academy of Sciences, 840 05 Bratislava, Slovakia
2	Department of Biochemistry, Faculty of Natural Sciences, Comenius University, 84215 Bratislava, Slovakia
3	Department of Functional Organization of Biomembranes, Institute of Experimental Medicine, Academy of Sciences of the Czech Republic, 142 20 Prague, Czech Republic
*	Corresponding authors at maria.balazova@savba.sk and jan.malinsky@iem.cas.cz
S-1

## Slide 2
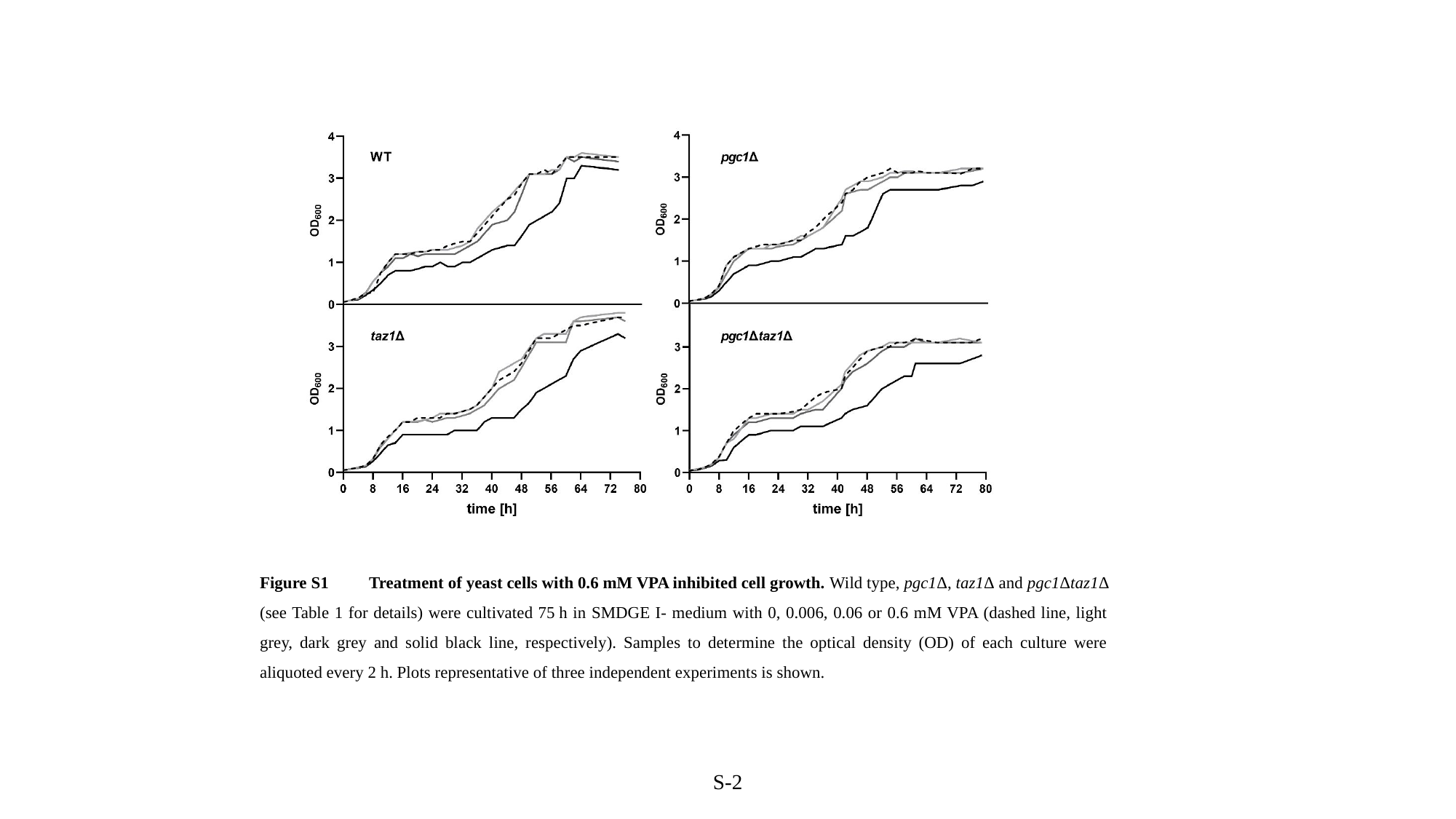

Figure S1	Treatment of yeast cells with 0.6 mM VPA inhibited cell growth. Wild type, pgc1Δ, taz1Δ and pgc1Δtaz1Δ (see Table 1 for details) were cultivated 75 h in SMDGE I- medium with 0, 0.006, 0.06 or 0.6 mM VPA (dashed line, light grey, dark grey and solid black line, respectively). Samples to determine the optical density (OD) of each culture were aliquoted every 2 h. Plots representative of three independent experiments is shown.
S-2

## Slide 3
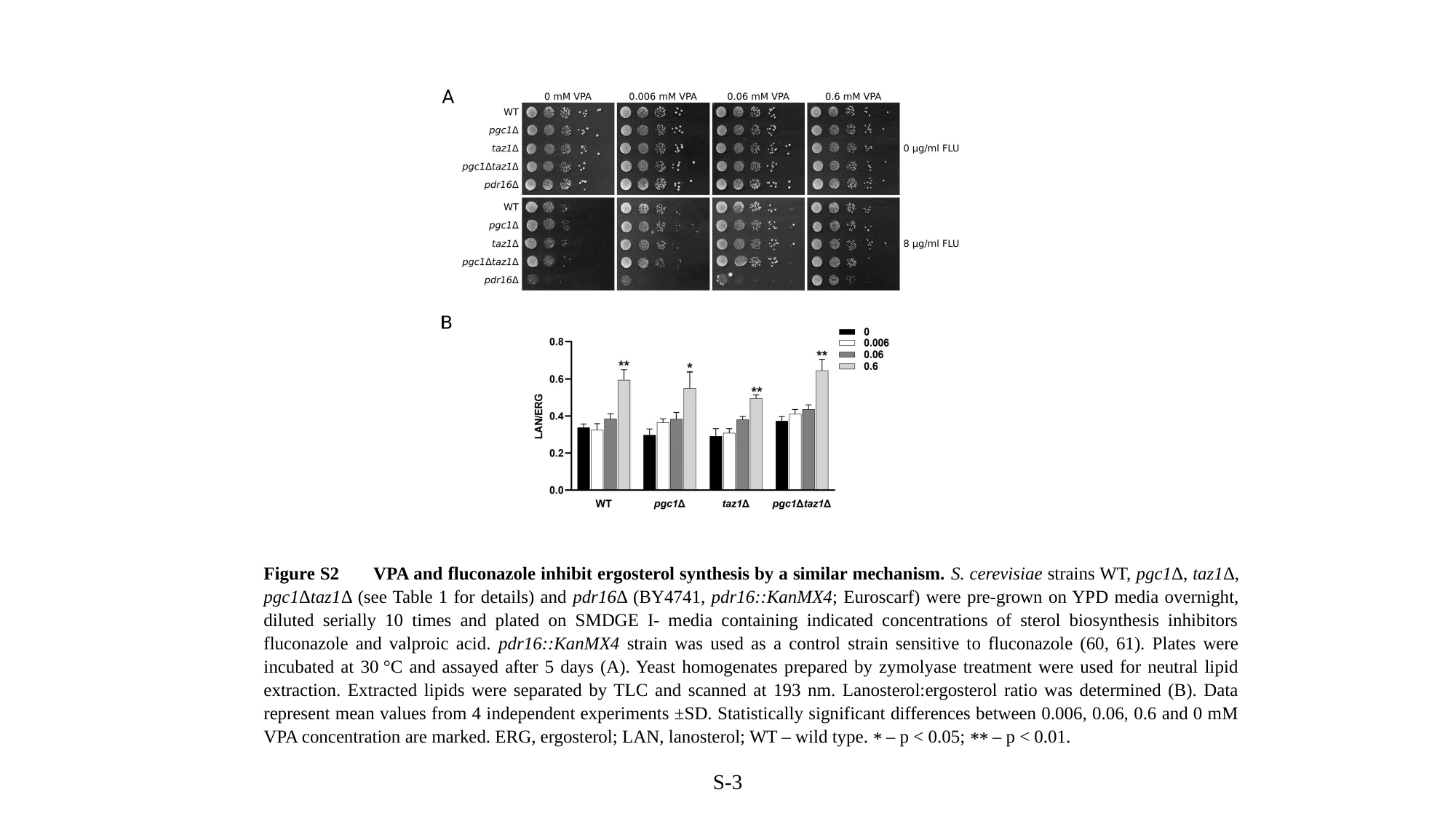

Figure S2	VPA and fluconazole inhibit ergosterol synthesis by a similar mechanism. S. cerevisiae strains WT, pgc1Δ, taz1Δ, pgc1Δtaz1Δ (see Table 1 for details) and pdr16Δ (BY4741, pdr16::KanMX4; Euroscarf) were pre-grown on YPD media overnight, diluted serially 10 times and plated on SMDGE I- media containing indicated concentrations of sterol biosynthesis inhibitors fluconazole and valproic acid. pdr16::KanMX4 strain was used as a control strain sensitive to fluconazole (60, 61). Plates were incubated at 30 °C and assayed after 5 days (A). Yeast homogenates prepared by zymolyase treatment were used for neutral lipid extraction. Extracted lipids were separated by TLC and scanned at 193 nm. Lanosterol:ergosterol ratio was determined (B). Data represent mean values from 4 independent experiments ±SD. Statistically significant differences between 0.006, 0.06, 0.6 and 0 mM VPA concentration are marked. ERG, ergosterol; LAN, lanosterol; WT – wild type. ⁎ – p < 0.05; ⁎⁎ – p < 0.01.
S-3
